# Incisional hernia repair using component separation with perforator preservation and onlay mesh: A pilot study

**DOI:** 10.1007/s10029-026-03644-4

**Published:** 2026-03-31

**Authors:** Yasser A. Orban, Yasser Baz, Yasmine Hany Hegab, Reham Zakaria, Ibrahim A. Heggy

**Affiliations:** 1 Department of General Surgery, Faculty of Medicine, Zagazig University, Zagazig, Sharqia, Egypt; 2 Department of General Surgery, Faculty of Medicine, Helwan University, Cairo, Egypt

**Keywords:** Component release, incisional hernia, component separation, perforator

## Abstract

**Introduction:**

Incisional hernia (IH) is a common complication after abdominal surgeries, whether conventional open or laparoscopic. Despite many efforts to reduce the incidence of IH, there is still a lack of consensus concerning the best approach for its prevention and repair. This pilot study evaluates a technique integrating perforatorpreserving anterior component separation with onlay mesh reinforcement at the midline and lateral external oblique incisions, addressing persistent challenges in tension management, vascular preservation, and recurrence reduction post-abdominal surgery.

**Patients and methods:**

This is a prospective pilot study conducted from January 2023 to January 2024 to evaluate the repair of large midline incisional hernias in 17 patients who underwent previous vertical midline incisions due to various indications. We used anterior component separation with perforator preservation along with onlay mesh reinforcement.

**Results:**

All cases underwent only mesh fixation. There were no intraoperative complications reported. The reported postoperative complications were wound seroma (41.2%), superficial wound ischemia (5.9%), and wound seroma with superficial wound ischemia (5.9%), while no complications were encountered in the rest of the cases (47.1%). There were no reported cases with deep wound ischemia and/or wound dehiscence.

**Conclusion:**

The anterior component separation technique with perforator preservation is a feasible and effective method for treating large incisional hernias with difficult midline closures. When paired with onlay mesh reinforcement for both the midline and lateral releasing incision, it provides satisfactory outcomes, low recurrence rates, and a manageable complication profile. The technique of the procedure should be tailored to each patient's specific condition.

**Supplementary Information:**

The online version contains supplementary material available at 10.1007/s10029-026-03644-4.

## Introduction

Incisional hernia (IH) is a common complication after abdominal surgeries, whether open or laparoscopic. It occurs in about 15–20% of all laparotomies and 1–5% of all laparoscopic procedures, with an overall occurrence in 2–10% of all abdominal operations [1, 2].

The incidence of midline incisional hernia is about 12.8% [3] two years after a midline laparotomy. Approximately one-third of patients with an incisional hernia undergo surgical repair. Even after repair, recurrence rates range from 23 to 50%, with increasing rates of complications and re-recurrence after each subsequent unsuccessful repair [3, 4].

In massive ventral hernias where simple closure is impossible due to loss of domain or excessive tension, bridging mesh repair and component separation techniques (CST) provide options for managing such hernias. Bridging takes less time but has a higher rate of mesh bulging (pseudo-recurrence) because the abdominal muscles remain retracted and non-functional. In contrast, CST restore linea alba and abdominal wall continuity, enhancing long-term stability, making it the best standard for non-emergency large defects [5, 6].

Ramirez et al. [7] had originally described the component separation technique that is known as anterior component separation for achieving tension-free midline closure using autologous tissue. This is achieved by making a longitudinal incision in the external oblique aponeurosis approximately 1–2 cm lateral to the linea semilunaris. The posterior rectus sheath is also incised longitudinally 2 cm lateral to the linea alba. Incising the posterior rectus sheath allows the rectus complex (already loosened laterally by the external oblique release) to glide farther medially, independently of the posterior layers [8].

However, the classic anterior component separation (ACS) is associated with significant wound complications. The extensive subcutaneous dissection required to release the external oblique aponeurosis frequently disrupts the periumbilical perforating vessels supplying the skin of the anterior abdominal wall. This dissection contributes to high rates of major wound complications, including skin necrosis, infection, and dehiscence, which can compromise the repair and increase healthcare costs. To mitigate these risks, perforator-preserving ACS techniques have been developed [9, 10].

Standardized staging systems, such as the three-stage ventral hernia classification, provide a critical framework for procedure selection, surgical decision-making must remain patient-centric **(**Table 1**)** [11].

**Table 1:** The staging system for ventral hernia [12].

	Risk	Description
Stage I	Low SSO, low recurrence	♣ Clean, < 10 cm.
Stage II	Intermediate SSO, intermediate recurrence	♣ Clean, 10–20 cm. ♣ Contaminated, < 10 cm.
Stage III	high SSO, high recurrence	♣ Contaminated, > 10 cm. ♣ Any IH > 20 cm

*SSO* Surgical Site Occurrences like enterocutaneous fistula, hematoma, seroma surgical site infection, wound cellulitis, wound dehiscence

The recurrence rate after non-prosthetic primary fascial repair is unacceptably high, reaching up to 50%. Non-prosthetic repair should be reserved for small IH (< 2 cm) in patients who have no recurrence risk factors [12]. Tension-free repair using synthetic mesh has significantly lowered the recurrence rates (2–10%) and is the most often used repair technique. If the defect size exceeds 4 cm, the best method is tension-free mesh repair [13]. Mesh reinforcement has the potential to reduce the rate of recurrence without raising the incidence of serious perioperative complications [14].

The ACS technique provides effective midline fascial closure similar to posterior component separation and can surpass it, especially in the lower abdomen below the arcuate line; however, it has drawbacks, namely complications related to the extensive subcutaneous dissection that jeopardizes skin vascularity and the lateral releasing incision that weakens this area, making it a potential site for hernia recurrence [15–17].

The aim of this study was to assess the feasibility of a surgical approach for complex incisional hernia repair that integrates perforator-preserving tailored anterior component separation with onlay mesh reinforcement for the midline and the lateral releasing incision in the external oblique aponeurosis.

## Patient and methods

This is a prospective pilot study conducted from January 2023 to January 2024 to evaluate the repair of large midline incisional hernias in 17 patients who underwent previous vertical midline incisions due to various indications. We used anterior component separation with perforator preservation along with onlay mesh reinforcement. The study was carried out after IRB approval and in compliance with the Code of Ethics of the World Medical Association (Declaration of Helsinki) for experiments involving humans. Informed written consent was obtained from the patients. Patients aged from 18 to 70 years of both sexes who had clean IH of stages I, II, and III according to the staging system of ventral hernia were included.

The exclusion criteria included a BMI of 35 kg/m² or more, collagen disease, and uncontrolled diabetes or COPD. Patients with contaminated, parastomal hernias, stomas, or a history of aortobifemoral bypass were excluded. Patients who were unfit for surgery according to the ASA criteria or active smokers were excluded as well.

The patients were clinically evaluated through comprehensive history taking, including surgical and medical histories, as well as undergoing general and local clinical examinations.

We performed laboratory assessments (glycemic profiles, coagulation studies, liver function tests, renal function tests, and complete blood count). Imaging studies, including pelvi-abdominal ultrasonography and CT scans of the abdomen and pelvis, were utilized to determine the size, location, and number of hernia defects.

The patients underwent preoperative cardiopulmonary assessment and optimization, nutritional status, cessation of smoking for at least one month before operation for smokers, and control of blood glucose level for diabetics HbA1c (< 7). Staging of the hernia was done depending on the risk of infection and defect size in CT.

### Operative technique

Surgical procedures were performed with the patient in a supine position under general anesthesia. An elliptical incision including the previous scar was performed after skin preparation and draping.

**Fig. 1:**
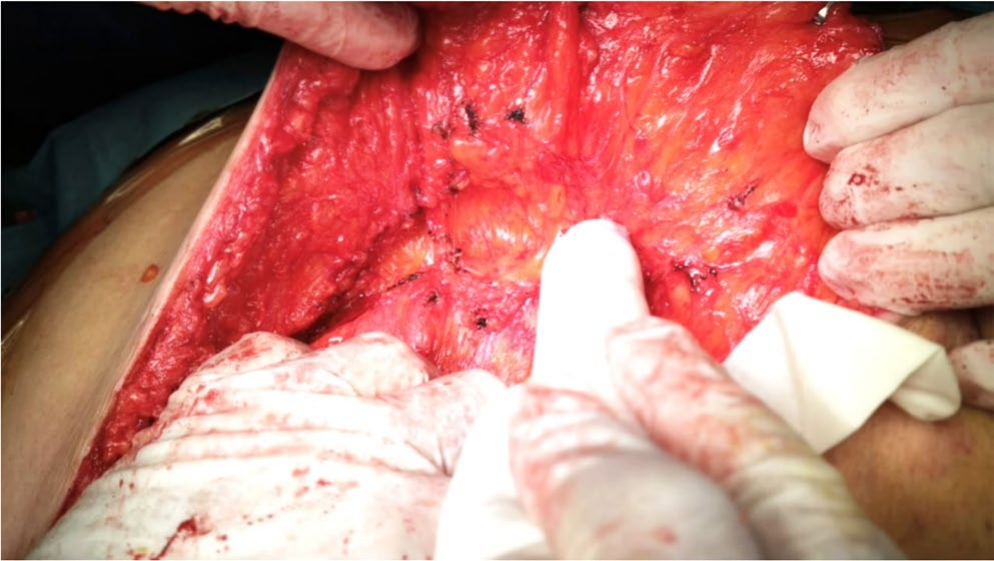
The use of the intraoperative hand held doppler to assess in the localization of the periumblical perforators

**Fig. 2:**
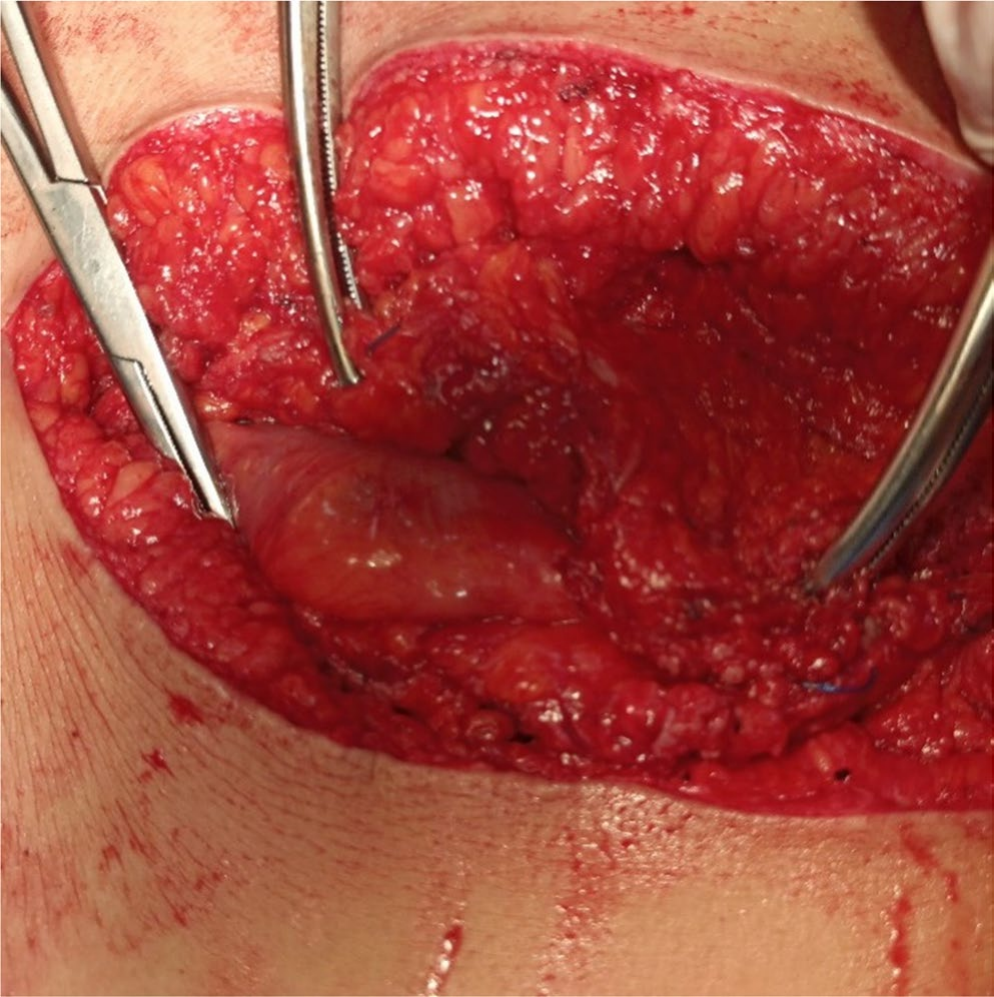
checking the tension at the midline while the fascia was approximated using clamps

Opening the sac and adhesiolysis were performed. Bilateral subcutaneous flaps were raised to allow mesh overlap for at least 8 cm after midline closure with great attention to preserve perforators. The periumbilical perforators were identified and preserved through intraoperative doppler-guided **(**Fig. 1**)** meticulous dissection with minimal surrounding subcutaneous fat. These perforators were located 2–5 cm above and below the umbilicus. The tension was evaluated while the fascia was approximated using clamps **(**Fig. 2**)**. Our goal was to overlap the fascia of both sides for about 1–2 cm when approximated together in the midline.

**Fig. 3:**
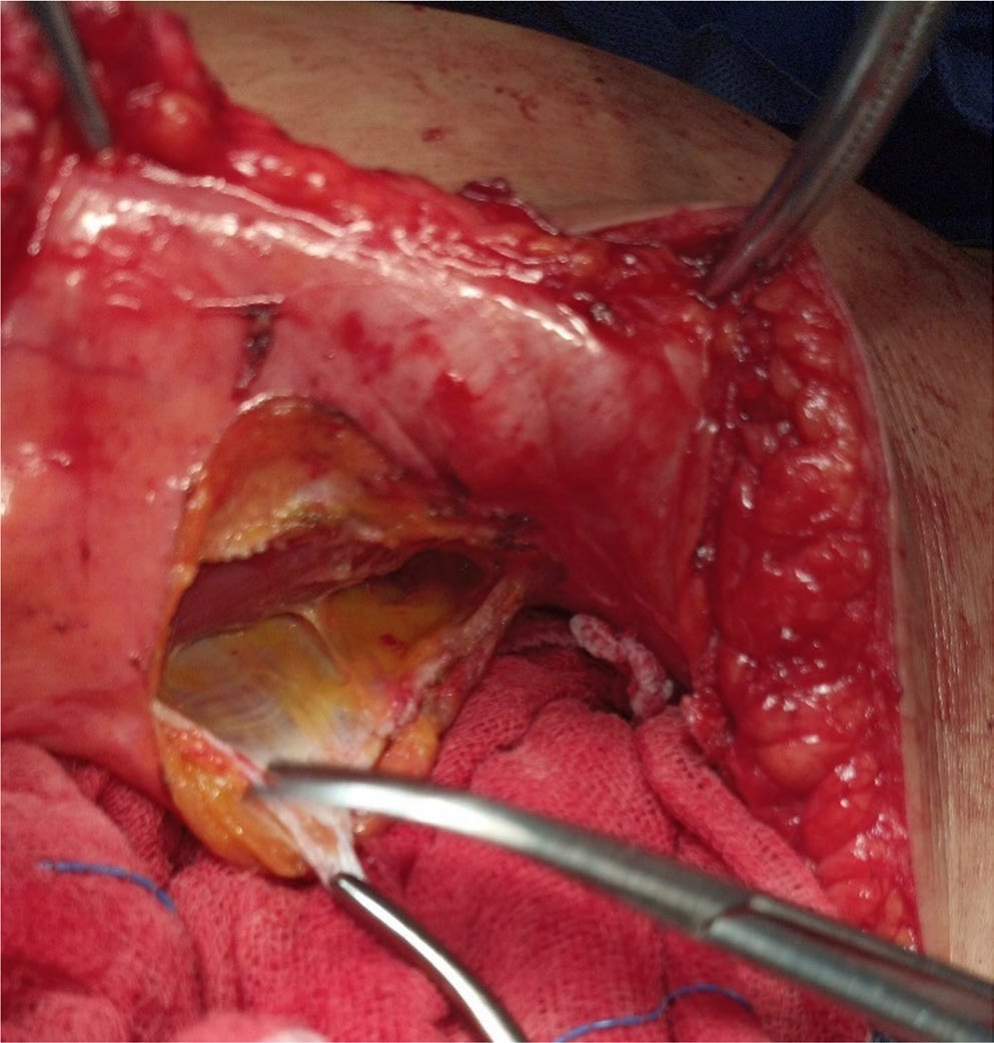
Posterior rectus sheath release

**Fig. 4:**
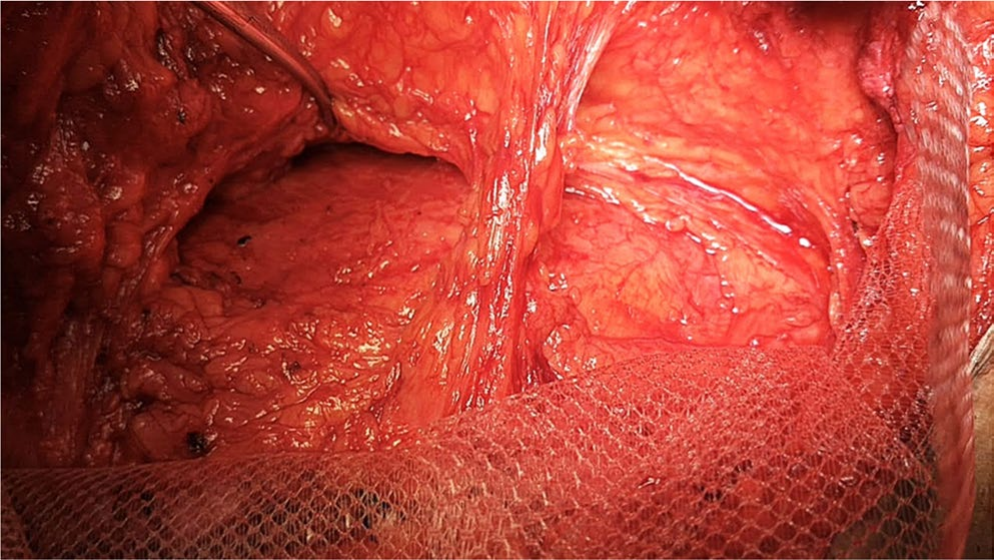
The preserved perforator with minimal subcutaneous fat. The lateral edge of the external oblique release is elevated by a clamp and the created space between the internal and external oblique muscles is demonstrated

If tension was present or there was a failure of midline approximation, selective myofascial advancement was then done. For myofascial advancement, we used classical, sequential components release as outlined by Ramirez (Anterior Component Separation), specifically tailored to release tension observed intraoperatively during midline closure [7]. We first performed a posterior rectus sheath unilateral release 1–2 cm lateral to the linea alba **(**Fig. 3**)** and reevaluated the tension at the midline. If tension remained, the posterior rectus sheath was released on the other side, and tension was reassessed at the midline. If bilateral posterior rectus sheath releases were done and tension was still present, the external oblique was unilaterally released 1–2 cm lateral to the linea semilunaris along the length of the abdominal wall. Subsequently, the space between the external and internal oblique muscles was established **(**Fig. 4**)**. Another external oblique release on the opposite side may be required if there was persistent tension.

**Fig. 5:**
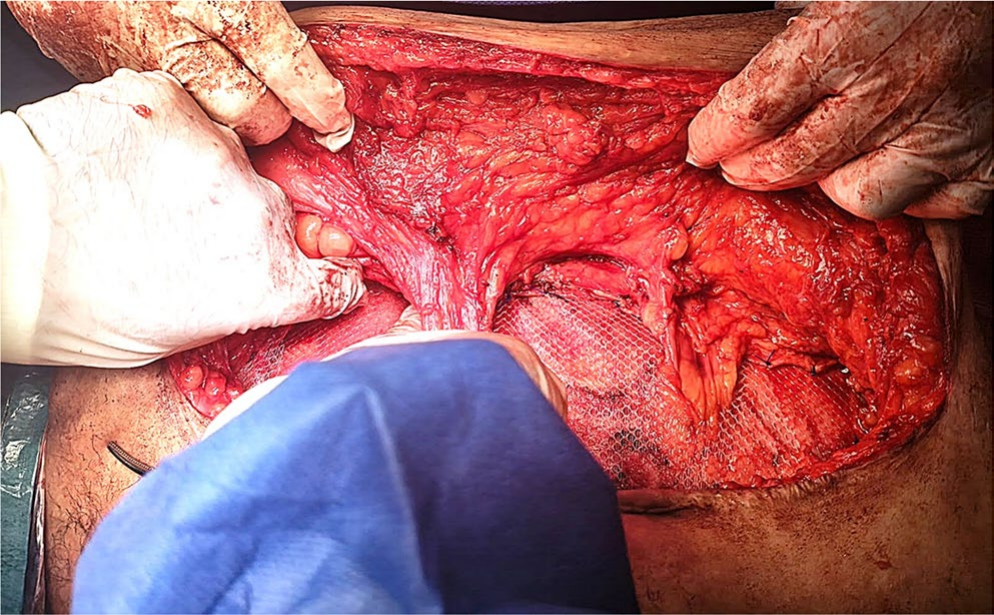
Cuts were made in the mesh to allow perforators to pass through without being compressed, and the mesh was deployed further laterally underneath the external oblique after its release

**Fig. 6:**
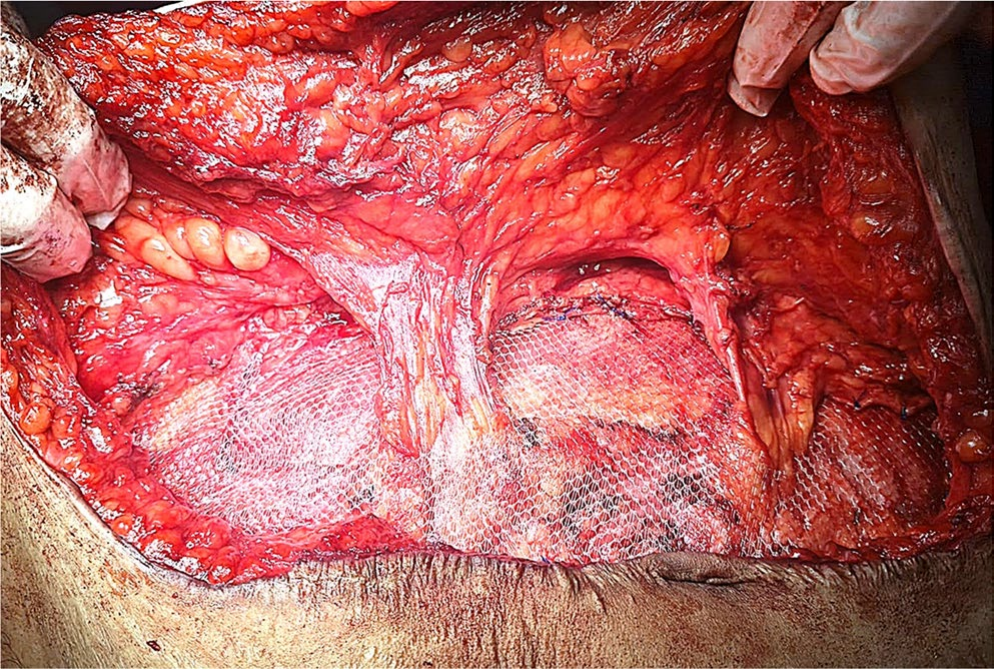
Mesh fixation

The hernial defect was then approximated at the midline with a running prolene^®^ -1- suture; thereby, recreation of the linea alba was done. A macroporous prolene mesh was then fixed over the fascial repair. The mesh was applied to reinforce the midline repair and overlapped laterally with cuts made in the mesh to allow the preserved perforators to pass through without being compressed. If external oblique release was performed, additional lateral mesh overlap was created between the external and internal oblique muscles **(**Fig. 5**)**. Several 2/0 prolene^®^ stitches were used to anchor the mesh with attention to fix the mesh at the repair site and the cut edges of the external oblique release **(**Fig. 6**)**.

Two suction drains were placed in the subcutaneous space. The subcutaneous tissue was approximated using 3/0 polyglactin sutures. The skin incision was closed by subcuticular 2/0 prolene^®^ reinforcement with interrupted stitches.

Diabetic patients were given regular insulin based on blood glucose checks every 6 h. Patients were encouraged to mobilize early while wearing an abdominal binder and to start oral fluids as soon as intestinal sounds became audible. The wound was dressed every other day. The drain was removed when it drained 30 cc or less of serous fluid per day.

**Fig. 7:**
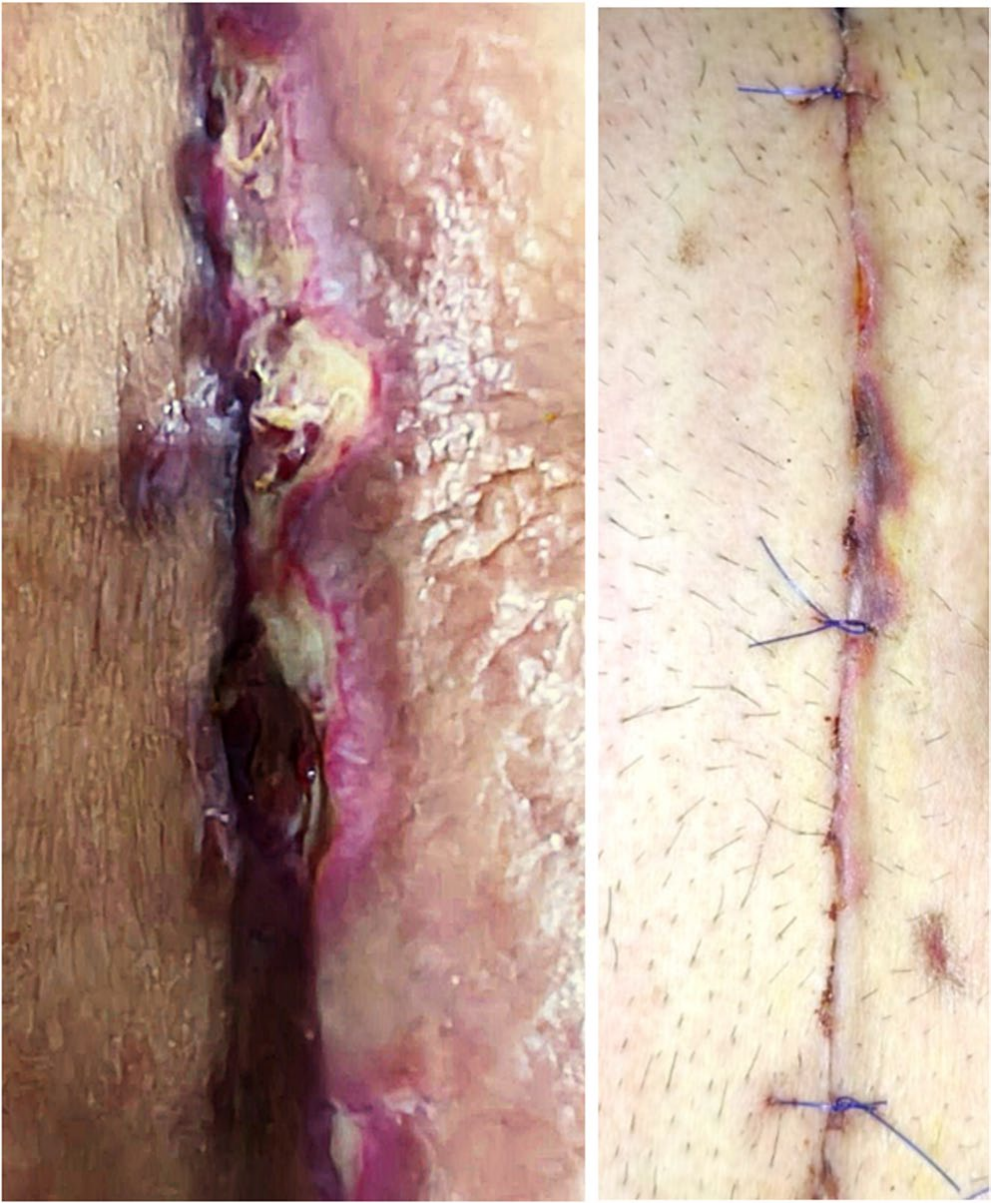
The appearance of postoperative superficial wound ischemia

Participants were monitored for at least six months: weekly for the first month, then every two weeks for two months, and then monthly. Follow-up visits were conducted at the outpatient clinic to examine the surgical site for signs of hematoma, seroma, infection, wound dehiscence, fistula formation, or flap alterations like deep or superficial ischemia **(**Fig. 7**)**, as well as for removal of the drains and stitches and identification of hernia recurrence.

## Results

Seventeen patients were involved in this study, seven males and 10 females, with a mean age of 48.8 ± 8.9. The main complaint was abdominal protrusion at a site of previous abdominal operation, 52.9% **(**Tables 2 and 3**)**.

All the patients had previous abdominal surgery **(**Table 2**)**. The most frequent laparotomy was exploration for intraabdominal sepsis (7 patients). Other causes for the previous laparotomy were exploration for abdominal trauma (2 patients), splenectomy for hypersplenism (5 patients), and colectomy for colon cancer (3 patients).

52.9% of patients presented with comorbidities, including diabetes mellitus, smoking habits, and hypertension **(**Table 2**)**.

All patients presented with a large abdominal wall defect ranged between 7 and 12 cm, measured on CT in the longest transverse dimension **(**Table 3**)**. According to the IH staging system, which is based on the dimensions of the hernial defect observed in CT images and the associated infection risk, stage I was found in 47.1% of patients, and stage II was identified in 52.9% of cases.

The most frequent technique employed in this study was bilateral posterior rectus sheath release combined with bilateral external oblique release, accounting for 52.9% of cases, followed by bilateral posterior rectus sheath release with unilateral external oblique release at 29.4%, then bilateral posterior rectus sheath release alone which was performed in 17.6% of cases. All cases underwent onlay mesh fixation **(**Table 2**)**. There were no intraoperative complications reported.

**Table 2:** shows preoperative data, procedures, and postoperative complications

			Frequency	Percent
Sex	Male		6	35.3
	Female		11	64.7
Stage	Stage I		8	47.1
	Stage II		9	52.9
Symptoms	Abdominal bulge	Yes	17	100.0
	Skin changes	NO	17	100.0
	Abdominal pain	Yes	17	100.0
	Unsatisfactory cosmetic appearance	NO	8	47.1
		Yes	9	52.9
	Abdominal bulge & pain		8	47.1
	Abdominal bulge, pain & unsatisfactory cosmetic appearance		9	52.9
Indication of previous laparotomy	Exploration for radical colectomy		3	17.6
	Splenectomy for hypersplenism		5	29.4
	Exploration for perforated appendicitis		3	17.6
	Exploration for perforated peptic ulcer		4	23.5
	Exploration for trauma		2	11.8
Comorbidities	No comorbidity		8	47.1
	Smoking		2	11.8
	Hypertension		3	17.6
	Diabetes		3	17.6
	Hypertension & diabetes		1	5.9
	Total comorbidities		9	52.9
Procedure	Bilateral PRR + onlay Mesh		3	17.6
	Bilateral PRR + Unilateral EOR +onlay Mesh		5	29.4
	Bilateral PRR + Bilateral EOR +onlay Mesh		9	52.9
Complications	No complications		8	47.1
	Wound seroma		7	41.2
	Superficial wound ischemia		1	5.9
	Deep wound ischemia and wound dehiscence		0	0
	Wound seroma and superficial wound ischemia		1	5.9

*PRR* Posterior Rectus Release

**Table 3:** showed age, defect size, operative time and hospital stay

	*N*	Minimum	Maximum	Mean	Std. deviation
Age	17	36	63	48.8	8.9
Defect size	17	7	12	9.6	2
Operative time	17	81	134	103	14.7
Hospital stays	17	2	12	5.5	2.9

*Std* standard, *N* number

The reported postoperative complications (Table 2) were wound seroma (41.2%), superficial wound ischemia (5.9%), and wound seroma with superficial wound ischemia (5.9%), while no complications were encountered in the rest of cases (47.1%). There were no reported cases with deep wound ischemia and/or wound dehiscence.

Most seroma patients were asymptomatic and detected via ultrasonography during the follow up. These cases were managed conservatively, with complete spontaneous resolution observed within 2 to 4 months. Only three patients presented with a symptomatic bulge, which were effectively treated with repeated ultrasound-guided aspiration. Additionally, cases of superficial wound ischemia were managed non-operatively with repeated wound dressing, achieving complete healing without the need for surgical intervention.

## Discussion

Repairing a huge IH with substantial loss of domain is technically challenging and associated with increased morbidity, mortality, and recurrence rates [18]. The component separation technique described by Ramirez and his colleagues is valuable for the repair of large defects of the abdominal wall and allows tension-free closure of the myofascial layers of the abdominal wall [7].

Despite the effectiveness of the anterior component separation procedure for abdominal closure in large incisional ventral hernia, the technique has its drawbacks. It requires significant lateral dissection and mobilization of the skin and subcutaneous tissue to expose the retracted external oblique aponeurosis. The presence of a large dissected area can result in hematoma, seroma, and infection. The skin and subcutaneous tissue mobilization for a wide area can also endanger the blood supply, resulting in skin necrosis and wound dehiscence. Skin necrosis that occurs in the anterior component separation technique is related to excessive dissection in the musculocutaneous plane, causing perforator vessel damage, and tight skin closure by hindering skin blood supply [19].

In our study, the observed seroma formation rate exceeds the typical range reported in recent literature, where incidence rates for open ACS generally fluctuate between 15% and 30% [20]. Most seroma patients in our cohort were asymptomatic discovered during the follow up by ultrasonography.

Superficial wound ischemia is defined as a localized failure of blood flow to the skin edges and superficial tissues of surgical incision and often causes minor injuries like blisters and delayed wound healing [21]. Superficial ischemia is a common complication in hernia repairs, particularly when using onlay mesh [19]. All patients with superficial wound ischemia were managed conservatively by repeated dressing. There was no need for debridement, or surgical intervention. No wound dehiscence had been encountered.

There were no recorded cases of deep wound ischemia or dehiscence. As we adopted tailored techniques for each patient with perforators preservation. This coincided with a study conducted by Saulis et al. [22]. who reported skin ischemia or necrosis occurred in up to 20% of individuals who underwent surgery without preservation of periumbilical rectus abdominis perforator arteries, compared to only 2% in those with preservation.

Sukkar et al. [23] adopted a selected and limited lateral dissection starting from the defect edges, exposing the linea semilunaris and gaining access to the external oblique. The perforators in the periumbilical region were preserved by subcutaneous undermining above and below the umbilicus, resulting in a tunnel lateral to the perforators. Comparing this procedure to the old technique, the number of superficial wound complications was significantly reduced (from 20% to 2%) [22, 23].

Maas [24] also described a different modification by performing lateral skin incisions with direct access to the underlying oblique muscle release. This modification eliminates extensive subcutaneous mobilization, preserving the perforators. This approach didn’t gain popularity due to the rise of the endoscopically assisted techniques.

In this study, we selectively preserve the rectus perforator with minimizing the subcutaneous fat around. This approach enabled us to overlap the mesh laterally to add adequate reinforcement to the midline repair. Furthermore, the mesh can be overlapped further laterally beneath the external oblique and deployed between the external and internal oblique muscles if external oblique release had been performed. The procedure also reinforced the weakened site of the external oblique release. The lateral release incision is considered a potential site for lateral incisional hernia after anterior component separation [25]. We suppose that application of the mesh by this technique covers all potential sites of possible recurrence, either midline or lateral, that can’t be achieved with retrorectal mesh application.

Lowe et al. [26] reported a series of seven cases utilizing endoscopically inserted balloon dissectors within the subcutaneous space overlying the external oblique aponeurosis. This endoscopically assisted approach was associated with a 50% reduction in postoperative hospital stays for defects measuring up to 15 × 25 cm.

Although reherniation rate is still occurring because component separation was initially described to avoid the usage of prosthetic mesh [27]. The use of prosthetic mesh in IH repair, in combination with component separation, had reduced but not eliminated recurrence, with no significant increase in major or mild postoperative problems [28].

In the current study, postoperative hospital stays ranged from 2 to 12 days, consistent with Scheuerlein et al. [29].

To optimize the benefits of anterior component separation for the treatment of large midline IH, we used onlay mesh. Additionally, we considered that the procedure to fix an incisional hernia should be customized for the patient and the hernia’s characteristics. Thus, to achieve the best results, preoperative evaluation and preparation and a customized surgical strategy are necessary. No recurrence was noted throughout the six-month follow-up period.

## Conclusion

The anterior component separation technique with perforators preservation is a feasible and effective method for treating large incisional hernias with difficult midline closures. When paired with onlay mesh reinforcement for both the midline and lateral releasing incision, it provides satisfactory outcomes, low recurrence rates, and a manageable complication profile. The technique of the procedure should be tailored to each patient’s specific condition.

## Supplementary Information

Below is the link to the electronic supplementary material.

Supplementary Material 1

## Data Availability

The authors will make the raw data supporting the findings of this study available on reasonable request and without undue delay.

## Declarations

### Category of paper

Original article.
