# Supplementary material for: Incisional hernia repair using component separation with perforator preservation and onlay mesh: A pilot study
Source: Hernia. 2026 Mar 31;30(1):151. doi: 10.1007/s10029-026-03644-4 (PMC13038670; doi:10.1007/s10029-026-03644-4)
Supplement: Supplementary file 1 — Supplementary Material 1 [file 10029_2026_3644_MOESM1_ESM.docx]

We, the undersigned authors, hereby certify the following regarding the manuscript titled: “**Incisional Hernia Repair using Component Separation with Perforator Preservation and Onlay Mesh: A Pilot Study**”

**1. Ethical Approval**

The study was conducted in accordance with the ethical standards of the institution and the Helsinki Declaration. Informed written consent for treatment and publication of clinical details and images was obtained from the patient.

**2. Authorship**

All listed authors made substantial contributions to the conception, design, clinical management, data collection, and/or writing of this manuscript. Each author has read and approved the final version of the manuscript and agrees to its submission to the journal Esophagus.

**3. Originality and Exclusivity**

We affirm that this case report is original, has not been published previously, and is not under consideration for publication elsewhere.

**4. Conflict of Interest**

The authors declare no conflict of interest related to this work.

**Authors:**

Yasser A. Orban,

Yasser Baz

Yasmine Hany Hegab

Reham Zakaria

Ibrahim A.Heggy
